# Low soil phosphorus and high symbiotic fungal richness inhibits plant aboveground biomass in fragmented forests in China

**DOI:** 10.1038/s42003-025-08978-w

**Published:** 2025-11-18

**Authors:** Jing Guo, Liying Chu, Xuying Ye, William L. King, Jianbing Shao, Zhonghan Wang, Jinliang Liu, Chuwen Chen, Mingjian Yu

**Affiliations:** 1 College of Landscape Architecture, Zhejiang A&F University, Hangzhou, China; 2 College of Life Sciences, Zhejiang University, Hangzhou, China; 3 Forestry Bureau of Chun’an County, Hangzhou, China; 4 School of Biological Sciences, University of Southampton, Southampton, UK; 5 Xin’an River Ecological Development Group Corporation in Chun’an County, Hangzhou, China; 6 College of Life and Environmental Science, Wenzhou University, Wenzhou, China; 7 College of Forestry and Biotechnology, Zhejiang A&F University, Hangzhou, China

**Keywords:** Ecology, Plant sciences

## Abstract

Habitat fragmentation is a major threat to biodiversity, and it usually leads to microclimate variations. Habitat quality (e.g. nutrients and moisture) and fungal symbioses play important roles in plant growth and ecosystem productivity. However, the impact of habitat fragmentation on plant aboveground biomass (AGB) is unclear. We examined the soil nutrients, rhizosphere fungal richness, and the AGB of 10 woody plant species on 10 islands of the same age but varying in size and isolation, in a land-bridge island system of subtropical China. Here we show that island size, soil nutrients, and fungal symbioses are key factors driving plant growth patterns in a fragmented island system. Plant AGB is positively correlated with soil phosphorus (P) but negatively correlated with richness of symbiotic fungi, suggesting that P content is more impactful than fungal symbiosis on plant growth in subtropical fragmented forests. Across all islands, low soil P and high symbiotic fungal richness lead to decreased plant AGB on small islands. These findings highlight the critical role of environmental filtering in shaping plant development within island fragments.

## Abstract (web summary)

Habitat fragmentation reduces plant aboveground biomass on small islands via reduced soil phosphorus content and increased symbiotic fungal richness, highlighting the critical role of environmental filtering in fragmented forests.

## Introduction

Habitat fragmentation is a leading cause of biodiversity loss due to the simultaneous impacts on habitat area, connectivity and quality^1–3^. With rapid urbanization and land use change in recent decades, anthropogenic habitat fragmentation has become a common phenomenon and poses substantial threats to biodiversity^4,5^. Plant biomass and production are important properties for ecosystem functions^6^. However, most studies focus on the relationship between species number and the increasing area of a sampled island, which is known as the island species‒area relationship^7–9^. But, the mechanisms driving plant biomass variation among islands are unclear.

Soil nutrient levels and microbial symbionts are critically important for plant growth and ecosystem functions^10,11^. Fungal symbionts can acquire mineral nutrients for host plants, in return, they receive photosynthetically-derived carbohydrates from the host^12,13^. Generally, mycorrhizal fungi provide the greatest host-benefit in nutrient-poor environments. The dependence of plants on mycorrhizal symbiosis often decreases in nutrient-rich environments to reduce unnecessary carbon investment in mycorrhizal fungi^11,14^. Higher nutrient availability and lower host mycorrhizal colonization have been found to promote plant development and reproduction with increasing forest fragment size^15^. Therefore, developing a predictive framework for fungi-plant interactions among islands is vital for understanding ecosystem functions such as production and nutrient cycling.

Environmental filtering has been defined as the process maintaining species capability of surviving and persisting selected by abiotic environment in a given location^16^. Microorganisms live with macroorganisms to form complex biotic interactions^17,18^, they display spatial biogeographic patterns with the alteration of the local environmental conditions of remnant habitats or the increase of dispersal efforts to reach isolated habitats^9,19^. The variation in the diversity and composition of soil microbial communities may further affect plant growth and development^20,21^. In recent studies on pine rockland fragments, variations in microbial community structure among fragments had a considerable impact on plant performance and resource allocation, with higher plant community productivity being linked to greater microbial diversity in larger, more connected fragments^22,23^. More knowledge on the functional groups of microbiomes is needed to fully understand how habitat fragmentation affects plant development.

Fungal diversity generally decreases with microhabitat alterations resulting from habitat fragmentation. Moreover, this trend may vary among different functional groups^24^. Fungal functional groups are generally characterized into symbiotrophs, saprotrophs, and pathotrophs^25^. Because of their distinct nutrient acquisition strategies, each of these groups responds differently to various habitat conditions and therefore can be affected by different ecological factors^26^. Approximately 80% of terrestrial plant species establish symbiotic relationships with mycorrhizal fungi, which then promotes nutrient acquisition and resistance to abiotic stress and pathogens^12,27^. Therefore, symbiotic fungi are critical for plant growth and community structure^28^. However, few empirical studies have focused on the influence of the symbiotic fungal traits on plant biomass in fragmented forests. The species richness of mycorrhizal fungi can be associated with both increases^10,29^ and decreases^30,31^ in biomass of individual plant species or the plant community. These complex relationships between mycorrhizal fungal richness and plant biomass production might be due to differences between the identity and functional attributes of the organisms involved, as well as the environmental conditions, especially soil nutrient limitation^20,31^. To the best of our knowledge, the relationships between island attributes, symbiotic fungal richness, and plant biomass have not been studied in fragmented forests.

According to the theory of island biogeography^32^, a reduction in habitat area usually results in a higher extinction rate, whereas a reduction in habitat connectivity results in a lower immigration rate, both of which lead to a decline in biodiversity. In addition, habitat quality often decreases with decreasing fragment area due to intense edge effects in smaller habitats, thus affecting biodiversity^4,33^. Despite the important roles of soil fungi in plant development and forest ecosystem functioning, fungi are less studied in fragmented landscapes than plants and animals^24,34^. Trophic groups of fungi can be particularly sensitive to altered microhabitat conditions with land use changes^35,36^. Previous research has shown significant variations in dispersal capacity among root-associated symbionts, and the spore loads of some highly dispersive species decreased rapidly with increasing distance from source patches^37^. Meanwhile, P-deficient soils were found to contain more spores but lower diversity of arbuscular mycorrhizal fungi compared to P-rich soils^38^. The richness of arbuscular mycorrhizal fungi showed increase with ecosystem degradation in a recent study^39^. In general, symbiotic fungi in newly established habitats can be strongly affected by habitat area and spatial isolation because of environmental filtering (or abiotic selection) and dispersal limitation, while the specific outcomes are complex and uncertain^38,39^.

In this study, we explored how plant aboveground biomass (AGB) varied with soil nutrient and fungal traits on land bridge-islands in the Thousand Island Lake (TIL), Zhejiang Province, eastern China. We analyzed the effects of fungal symbiosis on plant AGB in the context of the biogeographical island theory. We made the following hypotheses: (1) Although there is strong variation in AGB and rhizosphere fungal traits among different plant species, fungal richness around these plants would always be greater on larger islands following the island biogeography theory of species migration and extinction^32^, (2) plant AGB would be greater under phosphorus-rich conditions in subtropical forests where plant growth is generally limited by phosphorus (P), and the dependence of plants on fungal symbionts would decrease in P-rich areas, thereby reducing mycorrhizal-associated carbon expenditures^13,40^, (3) habitat fragmentation would affect plant AGB via altered soil P and symbiotic fungal groups due to the roles of environmental filters in forest fragments^23,41^.

Our results revealed that in contrast to the positive species-area relationship of overall fungi, the richness of symbiotic fungi was negatively correlated with island area. In this subtropical region where plant growth is generally limited by P, plant AGB was positively correlated with soil P but negatively correlated with richness of symbiotic fungi, indicating the strong dependence of plant growth on P nutrients rather than fungal symbiosis. Across all islands, low soil P and high symbiotic fungal richness led to decreased plant AGB on small island. Therefore, our results confirmed the negative effect of habitat fragmentation on forest productivity.

## Results

### Variations in soil fungi and plant AGB

**Fig. 1:**
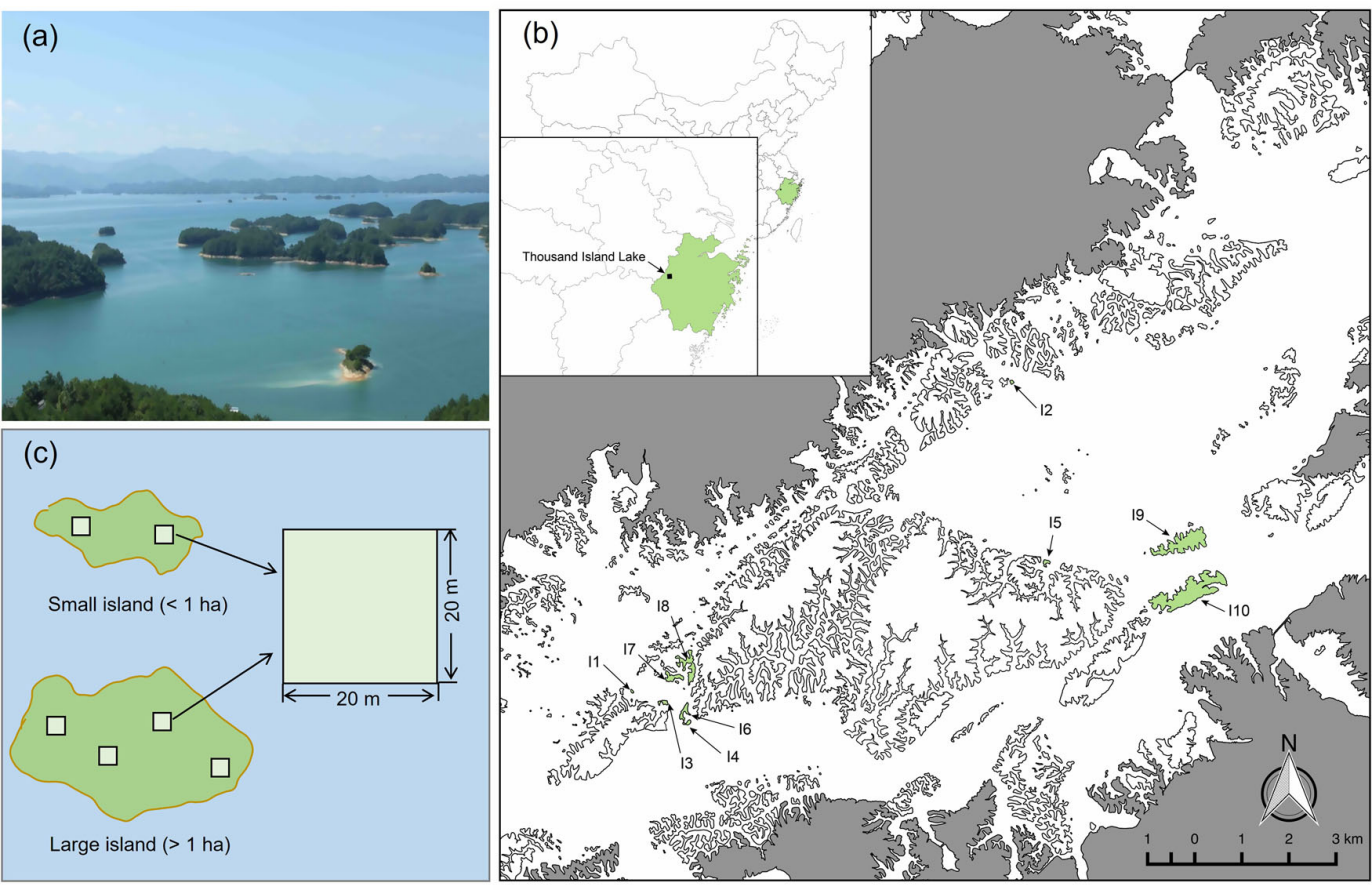
Island map of the Thousand Island Lake (TIL), eastern China. **a** The fragmented landscape of the TIL. **b** Locations of the 10 study islands in the TIL. The white areas in the main map represent water, and the gray areas represent the mainland. The study islands are shown in green. **c** Placement of 20 × 20 m investigating plots on each small (area < 1 ha) and large (area > 1 ha) island. The box represents the middle 50% of data, bounded by the first quartile and the third quartile. The median is the line that splits the box in two. Image in panel a was photographed by Mingjian Yu. Map in Panel b was created by Jing Guo and other co-authors. Image in panel c was drawn by Jing Guo.

**Fig. 2:**
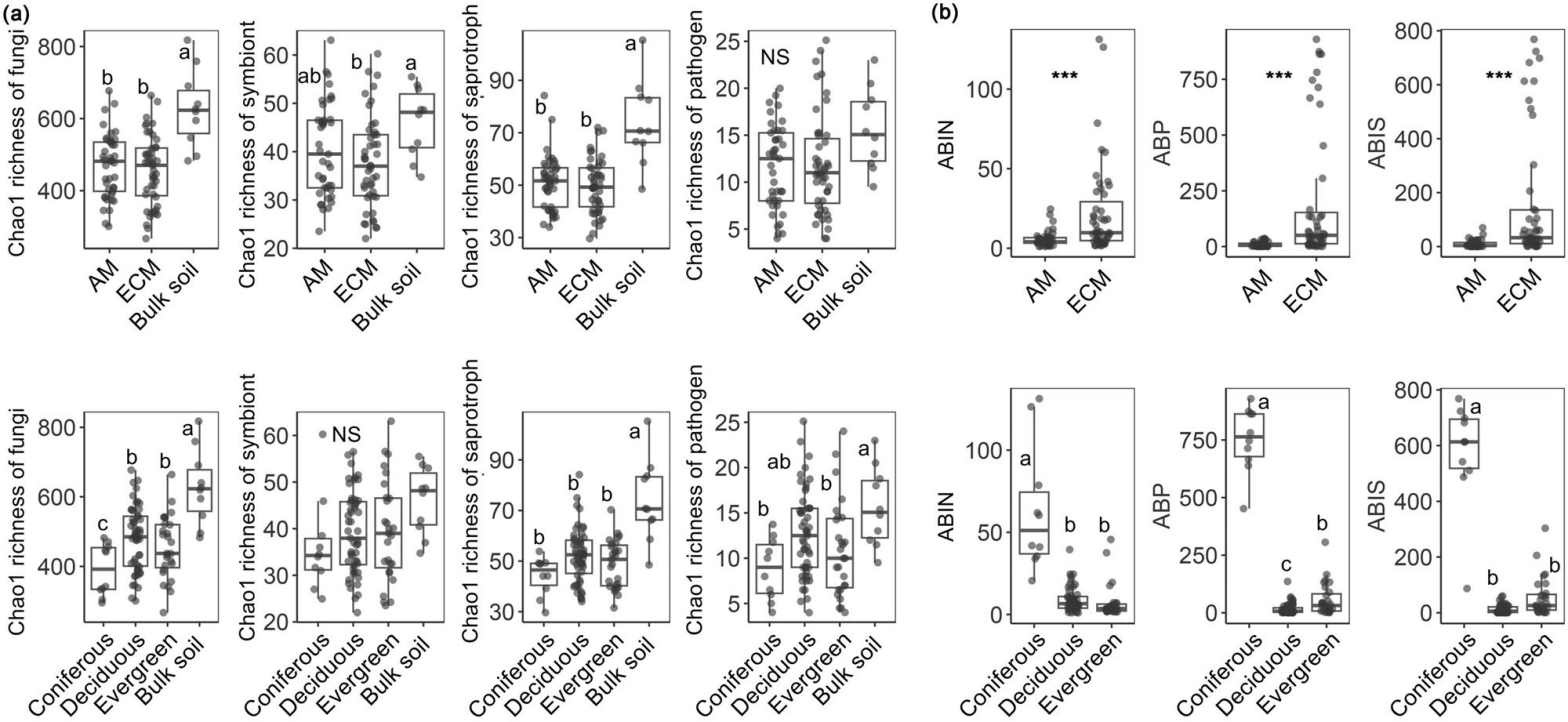
Variations in fungal richness and plant aboveground biomass between different plant functional types. **a** Fungal Chao1 richness in bulk and plant rhizosphere soils. **b** Plant aboveground biomass. Different letters or “*” represent significant differences between different types of plants or soils (*P* < 0.05). NS represents no significant difference (*P* > 0.05) between plant or soil types. ABIN, average aboveground biomass (AGB) per sampled individual plant; ABP, average AGB per individual of the target plant species in the 400 m^2^ plot; and ABIS, average AGB per individual of the target plant species on the entire island.

Through the analysis of all study sites, fungal richness (i.e., the richness of overall fungi) in the bulk soil was significantly greater than in the plant rhizosphere soil (*P* < 0.001; Figs. 1 and 2a; Supplementary Data 1). For the rhizosphere soil, significant differences in fungal richness were detected among the different leaf types (*P* = 0.02) but not between the different mycorrhizal types (*P* = 0.47). Fungal richness in the rhizosphere soil of coniferous trees (Masson pine) was significantly lower than that in the rhizosphere soil of deciduous broad-leaved species (Fig. 2a). Among the different leaf types of the plants, there was no significant difference in the richness of fungal functional groups, such as symbiotic, saprotrophic, or pathogenic fungi (all *P* > 0.05; Fig. 2a). Fungal composition did not vary clearly among the different plant species (Supplementary Fig. 1a).

There were significant differences for plant aboveground biomass (AGB) among different plant functional types (Fig. 2b). At both the individual (ABIN), 400 m^2^ plot (ABP), and island (ABIS) scales, the AGB of the ECM plants was significantly greater than that of the AM plants. In terms of leaf types, the AGB of coniferous trees was significantly greater than that of deciduous and evergreen broad-leaved species (Fig. 2b).

### Relationships between island spatial characteristics and soil fungi as well as plant AGB

**Fig. 3:**
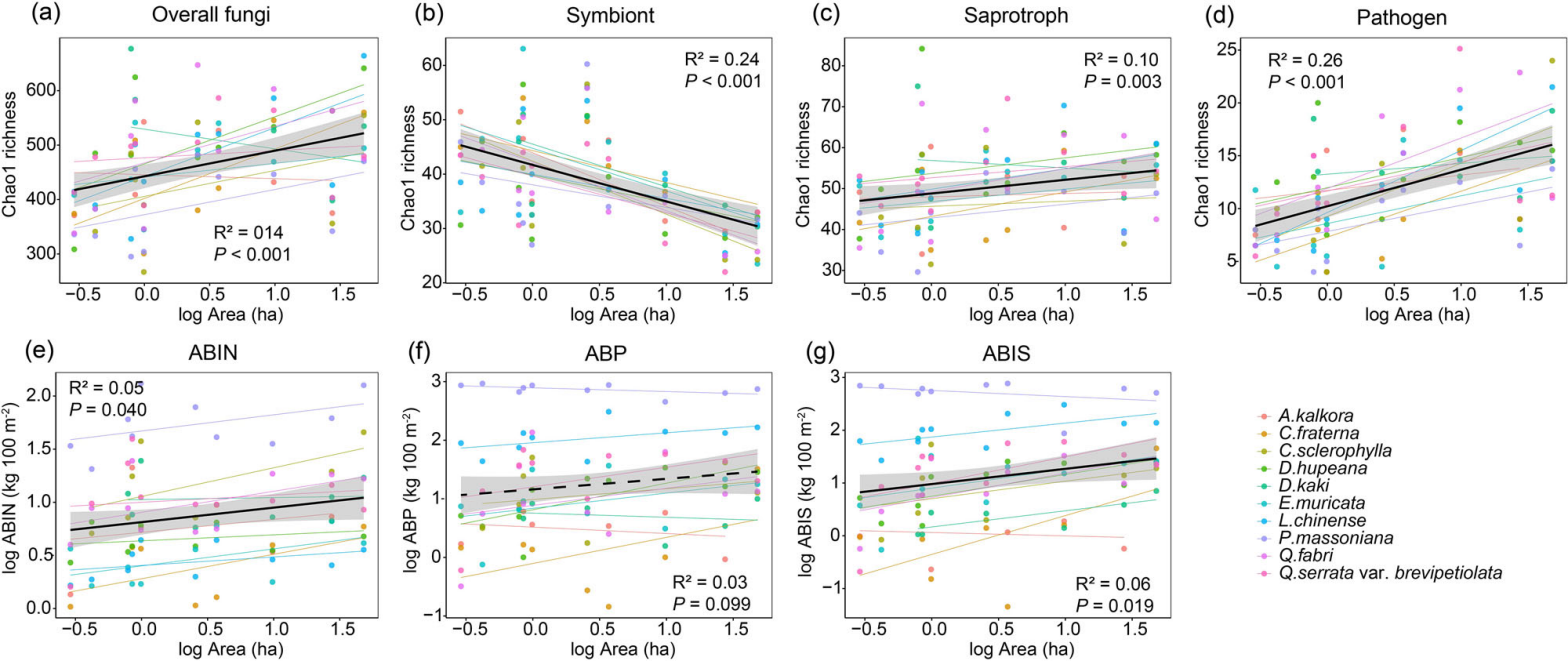
Relationships between island area and fungal richness as well as aboveground biomass. **a** Chao1 richness of overall fungi. **b** Chao1 richness of symbiont. **c** Chao1 richness of saprotroph. **d** Chao1 richness of pathogen. **e** ABIN, per sampled individual plant aboveground biomass (AGB). **f** ABP, average AGB per individual of the target plant species in the 400 m^2^ plot. **g** ABIS, average AGB per individual of the target plant species on the entire island. Different colors of the dotted lines represent fungal richness or average AGB of 10 plants. The black solid lines represent significant linear regressions (*P* < 0.05) and the black dashed lines represent marginally significant regressions (*P* < 0.10). The shading indicates the 95% confidence intervals around the regression.

Although there was strong variation in AGB and rhizosphere fungal richness between AM and EM-associated plants, and also strong variation among three leaf types, we detected consistent, significant correlations between island attributes (island area and isolation) and soil fungi as well as plant AGB (Fig. 3 and Supplementary Fig. 2). Specifically, fungal richness was positively correlated with island area (*P* < 0.001), with fungal richness in the bulk soil being more sensitive to island attributes variation than that in the rhizosphere soil of the plants (Fig. 3, Supplementary Table 1). Unlike the variation in overall fungi or other functional group, the richness of symbiotic fungi was negatively correlated with island area (*P* < 0.001; Fig. 3a–d).

A strong positive relationship between the overall fungal richness and island size was observed mainly in *Camellia fraterna, Dalbergia hupeana, Loropetalum chinense*, and *Pinus massoniana*. A strong, negative relationship between symbiotic fungal richness and island size was detected mainly in *Albizia kalkora*, *Diospyros kaki*, and *Quercus fabri*. Meanwhile, there was a significant relationship between island area and the richness of overall fungi and saprotrophs in the bulk soil (both *P* < 0.001; Supplementary Fig. 2). For fungal composition, we detected a relationship between island area and fungal community composition traits (Supplementary Figs. 1b and 2).

The plant AGB was positively correlated with the island area but negatively correlated with the symbiotic fungal richness in the rhizosphere soil (all *P* < 0.01; Fig. 3 and Supplementary Fig. 2). The linear correlations between the relative abundance of fungal functional groups and island spatial or plant biomass factors were negligible (Supplementary Fig. 2).

### Key drivers of plant aboveground biomass

**Fig. 4:**
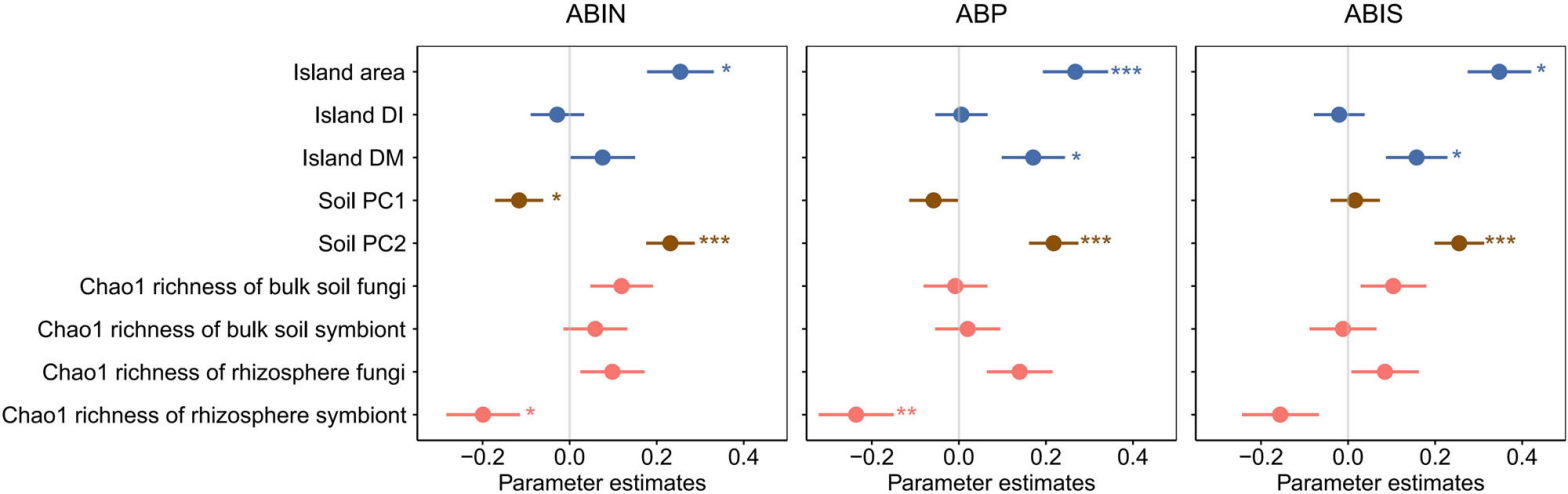
Relative effects of multiple predictors on plant aboveground biomass at the individual, plot and island scales. Three groups of predictors were used: (1) spatial factors, i.e., island area and isolation; (2) soil nutrient factors, i.e., PC1 (soil nitrogen) and PC2 (soil phosphorus) axis; and (3) soil fungal factors, i.e., species richness of fungi in bulk and rhizosphere soil. DI, distance to the nearest island; and DM, distance to the mainland. ABIN, average aboveground biomass (AGB) per sampled individual plant; ABP, average AGB per individual of the target plant species in the 400 m^2^ plot; and ABIS, average AGB per individual of the target plant species on the entire island. Values represent regression coefficients, and error bar represent 95% confidence intervals. *P < 0.05; **P < 0.01; and ***P < 0.001.

The first PC axis (PC1) explained 39.0% of the variance, was positively correlated with the soil pH, and was negatively correlated with the soil nitrogen content; thus, we refer to PC1 as the nitrogen axis. The second PC axis (PC2) explained 29.2% of the variance and was positively correlated with the soil phosphorus (P) content; thus, we refer to PC2 as the phosphorus axis (Supplementary Fig. 3 and Supplementary Table 2). The effects of island spatial and environmental variables and fungal diversity on plant AGB were generally consistent among the individual, 400 m^2^ plot, and island scales (Fig. 4). Compared with the other variables, the island area, soil P level (PC2) and symbiotic fungal richness in rhizosphere soil had stronger effects on plant AGB. Specifically, island area and soil P had significantly positive effects on plant AGB. The richness of the symbiotic fungal groups in rhizosphere soil negatively affected plant AGB.

### Linkage of plant biomass with island attributes

**Fig. 5:**
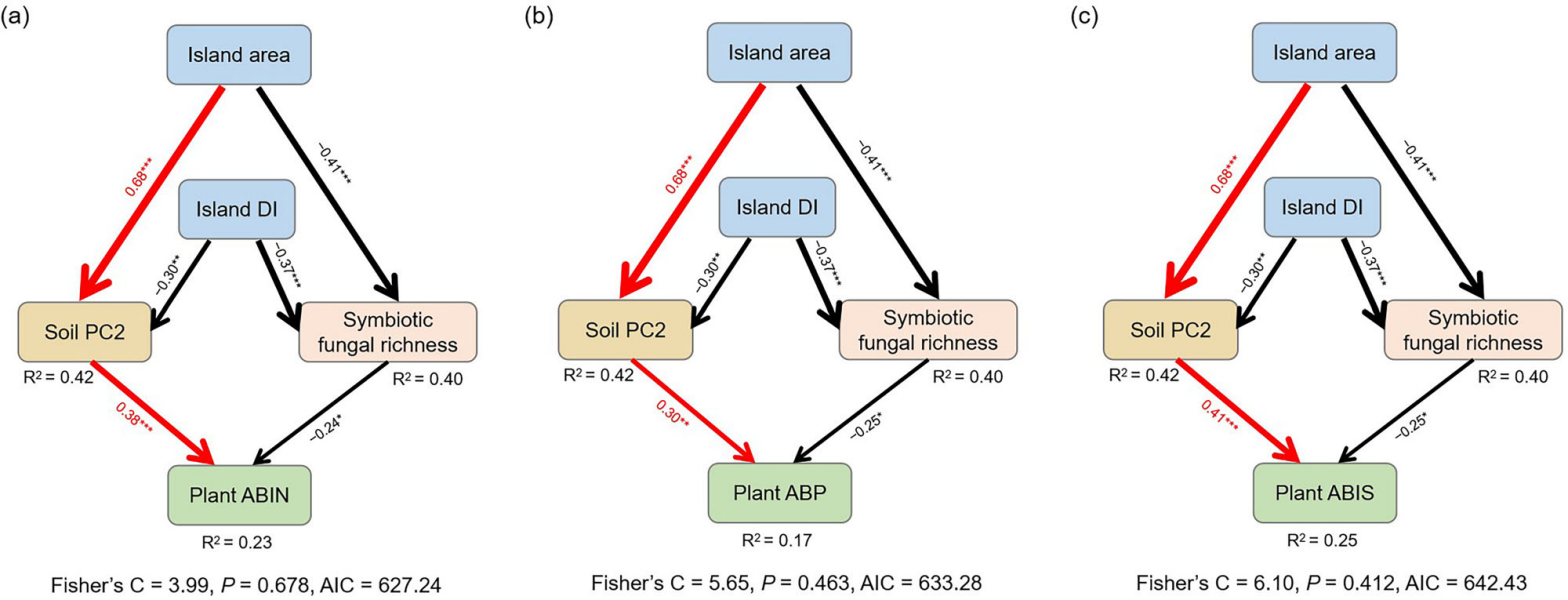
Structural equation model showing the pathways through which island spatial traits influence plant aboveground biomass. **a** Model of ABIN, average aboveground biomass (AGB) per sampled individual plant. **b** Model of ABP, average AGB per individual of the target plant species in the 400 m^2^ plot. **c** Model of ABIS, average AGB per individual of the target plant species on the entire island. Soil PC2 represents soil phosphorus level. DI, distance to the nearest island. The values associated with the arrows indicate standardized path coefficients. Red arrows indicate positive relationships and black arrows indicate negative relationships. The percentages associated with the response variables represent the variance explained by the model. *P < 0.05; **P < 0.01; and ***P < 0.001.

We used structural equation modeling (SEM) to link plant AGB to island spatial and soil nutrient variables as well as symbiotic fungal richness. SEM revealed that plant AGB and island spatial relationships were driven by the same mechanisms among the individual, 400 m^2^ plot, and island scales (Fig. 5). The plant AGB was driven by the island area and DI (distance to the nearest island) via altered soil P (PC2) and symbiotic fungal richness (Fig. 5). Compared with those on small islands, the higher soil P and lower richness of the symbiotic fungal groups on large islands increased plant AGB. Compared with the low-DI island, the lower soil P on the high-DI island reduced the plant AGB, whereas the lower richness of the symbiotic fungal group on the high-DI led to greater plant AGB (Fig. 5).

## Discussion

In the secondary forest fragments in the TIL (Thousand Island Lake), the biomass and rhizosphere fungi among the plant species that strongly differed in terms of leaf type and mycorrhizal type responded similarly to those in the island space (Fig.s 2 and 3). Compared with that of small, P-deficient islands, plant AGB increased with increasing soil P concentration on islands with large areas or low isolation (Figs. 4 and 5). Larger islands tend to have fewer edge habitats that experience abiotic stress, such as wind turbulence and soil leaching, thus producing more high-quality habitats that have P-rich soils^42,43^. In addition, soils in Chinese subtropical forests are generally deficient in P due to severe leaching under high-rainfall and high-temperature conditions^40,44^, which strongly limits plant growth and productivity^42^. On 10 islands in this study, the soil P concentration was only 0.21–0.31 mg g^-1^ (Supplementary Data 1). Therefore, higher P concentrations on larger islands can promote plant growth and thus increase AGB.

In addition to soil P conditions, we also identified associations between rhizosphere fungi, plant growth, and island fragmentation. The richness of symbiotic fungi decreased with the increase of island area (Fig. 3b). This result was inconsistent with those of other studies that reported insignificant^45^ or positive^46^ relationships between symbiotic fungal diversity and fragment area. Previous work has demonstrated positive species-area relationship of woody plants among islands in TIL^8^, thus the lower symbiotic fungal richness cannot be attributed to fewer plant species on the larger islands. One possible reason of lower symbiotic fungal richness is the strong competitive exclusion of dominant fungal taxa under nutrient rich conditions. With increasing island area and increasing P availability, increasing competition between symbiotic fungi for photosynthetic carbohydrates from host plants may result in a species poor community of symbiotic fungi^47^. Meanwhile, we observed a negative relationship between the proportion of symbiotic *Ascomycota* and *Glomeromycota* and large island area (Supplementary Fig. 2), which may partly indicate that island area affects symbiotic fungal richness via differential responses among symbiotic fungal phyla. Moreover, plant AGB was inversely related to the richness of symbiotic fungi (Fig. 4). This result supports our initial prediction and is consistent with some publications that reported the negative correlations between species richness of mycorrhizal fungi and plant growth in forest and grassland ecosystems^30,48^. Host plants of highly diverse symbiotic fungi may be more likely to reward less cooperative fungal taxa that truly act as carbon sinks, thus produce a negative AGB response in plant individuals^49^. Our findings support the low dependence of plants on symbiotic fungi on large, P-rich islands from the perspective of the species richness index. Besides, the influence of abiotic and biotic drivers on forest biomass dynamics may also exhibit spatiotemporal dependence on both abiotic and biotic drivers^50^.

Contrasting geographic distributions between symbiotic fungi and other functional group fungi have been reported in some previous studies^18,51,52^, which can be attributed to the strong competition between symbiotic fungi and saprotrophic fungi for soil resources^53^, and the great resistance of symbiotic fungi against pathogens^54,55^. Therefore, the distinct life histories among functional groups may generate variable biogeographic patterns. The mixed results that reflect functional-specific responses to fragmentation may be due to differences in host specificity, dispersal mode, and response to habitat conditions^56^. Specifically, the strong response of symbionts and pathogens to the island area gradient in the plant rhizosphere soil could be attributed to their close interaction with their host plant^20^. Given the lower slopes of species-area relationship for overall fungi in the plant rhizosphere soil relative to bulk soil (Fig. 3a, Supplementary Table 3), we hypothesize that root systems may provide a degree of resistance for soil fungi to habitat fragmentation at the measured scales^57^.

It is widely recognized that remoteness promotes biological invasions on islands^58,59^, thus may lead to niche packing of soil fungi on isolated islands. However, the dispersal limitation of most mycorrhizal fungal species^46,60^ and the negative effects of habitat isolation^61^ can cause a decrease in symbiotic fungal richness on remote islands. Another study reported the slight dispersal limitation of root-symbiotic fungi to distant islands^62^. In this study, symbiotic fungal richness was negatively correlated with habitat isolation (Fig. 5 and Supplementary Fig. 1), potentially suggesting an influence of dispersal limitation in structuring symbiotic fungi across the landscape. Nevertheless, plant AGB and associated habitat conditions were more significantly varied with island area than island isolation, which confirmed the strong effects of environmental filtering or abiotic selection process on plant productivity in island forest fragments. Similar results appeared in a study of plant-fungal interaction in fragmented forests^15^.

**Fig. 6:**
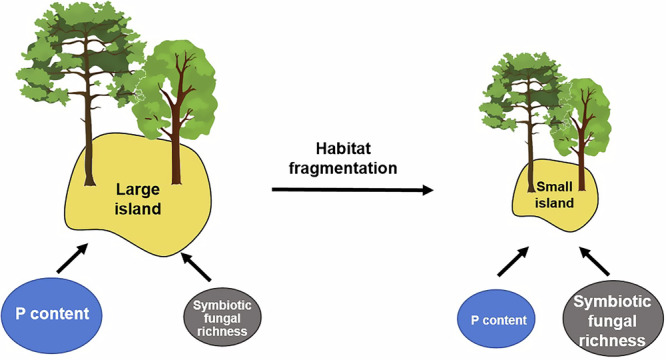
Conceptual framework of variations in soil nutrients, fungal richness and plant aboveground biomass among islands. Across different islands, plant aboveground biomass on small island was decreased due to lower soil P content and higher symbiotic fungal richness compared with large island. Within island, plant on small island rely more on fungal symbiosis due to low soil P level, whereas plants on large island rely more on soil P nutrient. Figure 6 was drawn by Jing Guo.

We showed that both plant AGB and fungal species richness were significantly driven by habitat fragmentation but through different mechanisms. Across all islands, island areas positively affected plant AGB via higher soil P levels but lower richness of symbiotic fungi. (Figs. 5 and 6), indicating the more strong effect of P content than fungal symbiosis on plant growth in Chinese subtropical forests^13,63^. Our results also suggest that within island, plants on small islands rely more on fungal symbiosis due to low soil P level; whereas plants on large island rely more on soil P nutrient, especially available P (Fig. 6). These patterns of plant development were consistent in spite of the strong variation in plant and rhizosphere fungal traits among the different plant species (Fig. 3 and Supplementary Fig. 2). Similarly in other temperate and subtropical forests, the reliance on mycorrhizal fungi was always reduced after nutrient additions across plants, despite their foraging strategies were affected by root morphology^64,65^. For fungi, species richness in the plant rhizosphere soil appeared to be driven by habitat fragmentation, with the richness of overall fungi being lower on small or isolated islands (Fig. 3a and Supplementary Fig. 4), indicating strong dispersal limitation and the extinction of fungi in island fragments^32,66^. Our results suggest the strong influence of environmental filtering and dispersal limitation on plant and fungal development, respectively, along fragment-size gradients. Further investigations on the impacts of habitat fragmentation on plant development should be explored to consider the roles of environmental filters, especially soil nutrients and fungal symbiosis. Moreover, future studies examining habitat fragmentation may consider doing temporal investigation on plant biomass, and using systematic, functional approaches to examine patterns between microorganisms and environmental data.

## Methods

### Study area

This study was conducted at the Thousand Island Lake (TIL) in Zhejiang Province, eastern China (Fig. 1). The TIL area is characterized by a subtropical monsoon climate, with local mean annual temperatures of 17.2 °C and mean annual precipitation of 1515 mm (China Meteorological Data Service Center, http://data.cma.cn/). The lake is an artificial hydroelectric reservoir formed by the construction of the Xin’anjiang Dam in 1959^67^. The lake has a total water area of 540 km^2^ and 1,078 land-bridge islands larger than 0.25 ha when the water reaches its highest level at 108 m a.s.l^68^. The forests on the fragmented islands (previous connected hill tops) were completely or almost clearcutting during the construction of the reservoir dam, and developed secondary succession up to now, thus they have a similar successional age. Currently, most forest areas on islands are dominated by Masson pine, *Pinus massoniana*, which is a common pioneer tree species in Chinese subtropical forest communities^69,70^.

### Sample collection

Ten islands ranging in area from 0.29–47.98 ha were chosen for this study (Fig. 1; Table 1). The distance from these islands to the nearest island (DI) varied from 12.66 m to 119.36 m, and the distance to the mainland (DM) varied from 1056.16 m to 4217.1 m (Table 1). Among these islands, five small islands covered less than 1 ha, and five large islands covered more than 1 ha. Small islands only had edge habitat and plant community structure of the island, and there was no interior plot can be chosen like large islands^71^. Ultimately, two replicate plots were established on each small island. On each large island, four replicate plots were established, with two plots on the edge and another two plots in the interior of the island (Fig. 1). Ten woody plant species were chosen for this study, with five associated with arbuscular mycorrhizal (AM) fungi and five associated with ectomycorrhizal (ECM) fungi (Table 2). The mycorrhizal type of each woody plant species was assigned on the basis of the morphological criteria according to published information^72,73^. In total, 30 plots (20 m × 20 m each) were established on all the islands, and 8–10 target plant species were included in each of these plots.

**Table 1:** Characteristics of the 10 study islands in the Thousand Island Lake region, southeastern China

Island ID	Latitude (N)	Longitude (E)	Perimeter (m)	Area (ha)	DI (m)	DM (m)
I1	29°30′38″	118°48′56″	203.60	0.29	83.39	3073.21
I2	29°34′07″	118°53′60″	245.27	0.42	28.36	1056.16
I3	29°30′29″	118°49′22″	402.52	0.79	13.28	2657.77
I4	29°30′16″	118°49′41″	488.39	0.85	18.41	2184.55
I5	29°32′01″	118°54′23″	542.99	0.99	59.81	4217.10
I6	29°30′22″	118°49′37″	1213.29	2.56	18.41	2199.38
I7	29°30′46″	118°49′30″	1364.00	3.70	12.66	2225.45
I8	29°30′59″	118°49′39″	3537.63	9.79	29.54	1901.72
I9	29°32′10″	118°55′56″	5622.30	27.49	47.82	1158.87
I10	29°31′30″	118°55′52″	7523.43	47.98	119.36	1066.10

DI, distance to the nearest island; and DM, distance to the mainland.

**Table 2:** Descriptions of the 10 woody species in the Thousand Island Lake region, southeastern China

Species	Family	Mycorrhizal type	Leaf habit
*Albizia kalkora*	Leguminosae	AM	Deciduous broad-leaved
*Camellia fraterna*	Theaceae	AM	Deciduous broad-leaved
*Dalbergia hupeana*	Leguminosae	AM	Deciduous broad-leaved
*Diospyros kaki*	Ebenaceae	AM	Deciduous broad-leaved
*Eurya muricata*	Theaceae	AM	Evergreen broad-leaved
*Castanopsis sclerophylla*	Fagaceae	ECM	Evergreen broad-leaved
*Loropetalum chinense*	Hamamelidaceae	ECM	Evergreen broad-leaved
*Pinus massoniana*	Pinaceae	ECM	Coniferous
*Quercus fabri*	Fagaceae	ECM	Deciduous broad-leaved
*Quercus serrata* var. *brevipetiolata*	Fagaceae	ECM	Deciduous broad-leaved

Roots and soil were sampled from April 20th–May 14th, 2022. One representative plant of each target species in a plot was selected for rhizosphere fungal estimation. Three to four root clusters were collected from shallow soil around the trunk of each selected plant within a 2 m radius. After gently shaking, the soil still adhering to the fine-root branches (e.g., first- and second-order roots) was considered as rhizosphere soil^74,75^. Rhizosphere soil around each plant were pooled together and mixed to form one sample. Moreover, five 0–10 cm deep soil cores (one in the center and four in each plot corner) were collected, mixed, and passed through a sieve of 2 mm mesh size to form one bulk soil sample. A total of 288 soil samples (258 rhizosphere soil and 30 bulk soil samples) were collected and stored at −80 °C for DNA extraction and sequencing. A total of 30 bulk soil samples were collected for measurement of physicochemical variables, including the soil bulk density, maximum water holding capacity (MWHC), soil pH, carbon (C), nitrogen (N), phosphorus (P), available N (ammonium and nitrate N) and available P.

Soil bulk density (g cm^−3^) and maximum water holding capacity (MWHC, g kg^−1^) were measured using the cutting ring method (LY/T 1215–1999)^76^. Soil pH was measured using a pH meter (SevenEasy S20K, Mettler Toledo, Greifensee, Switzerland), with water and soil in a 2.5:1 ratio by mass. For soil carbon (Soil C, mg g^-1^) and nitrogen (Soil N, mg g⁻¹) analysis, samples were ground and sieved (0.15-mm mesh) before measurement using an elemental analyzer (Vario MACRO Cube, Elementar, Langenselbold, Germany). Ammonium-N (NH₄⁺ − N, mg kg⁻¹) and nitrate-N (NO₃⁻ − N, mg kg⁻¹) were extracted with 2 mol/L KCl and quantified using a continuous flow analyzer (San + +, Skalar, Breda, Holland). Total soil phosphorus (Soil P, mg g^-1^) was digested with sulfuric acid–perchloric acid after grinding and sieving (0.15-mm mesh). Available phosphorus (mg kg^-1^) was extracted using ammonium fluoride and hydrochloric acid^77^. Both total and available P were measured by inductively coupled plasma optical emission spectrometry (Optima 8300, PerkinElmer, Waltham, MA, USA).

Woody plants were investigated in 2019. The number, height, diameter, and species of all free-standing plants with a diameter at breast height (i.e., 1.3 m above the ground) *≥*1 cm were recorded^68^. The AGB of each plant was calculated via published species-specific allometric equations with the predicted tree height (Supplementary Tables 3 and 4)^78^. The average AGB per plant for the sampled individuals (ABIN), each target plant species in the 400 m^2^ plot (ABP) and each target plant species on the island (ABIS) were calculated.

### DNA extraction and sequencing

Soil DNA extraction was performed via a TGuide S96 Magnetic Soil/Stool DNA kit (Tiangen Biotech (Beijing) Co., Ltd.). The DNA concentration was measured via a Qubit dsDNA HS assay kit and a Qubit 4.0 fluorometer (Invitrogen, Thermo Fisher Scientific, Hillsboro, ON, USA). The internal transcribed spacer 2 (ITS2) region of the rRNA gene was amplified with the ITS3F (5’-GCATCGATGAAGAACGCAGC-3’) and ITS4R (5’-TCCTCCGCTTATTGATATGC-3’) primer pairs^79^. The thermal cycling conditions for the ITS2 region were as follows: initial denaturation at 95 °C for 5 min, followed by 25 cycles of denaturation at 95 °C for 30 s annealing at 50 °C for 30 s, extension at 72 °C for 40 s, and a final step at 72 °C for 7 min. All PCR amplicons were purified via Agencourt AMPure XP Beads (Beckman Coulter, Indianapolis, IN) and quantified via the Qubit dsDNA HS assay kit and Qubit 4.0 fluorometer (Invitrogen, Thermo Fisher Scientific, Oregon, USA). The purified, pooled PCR products were subsequently sequenced on the Illumina NovaSeq 6000PE250 platform (Illumina, Santiago, CA, USA) at Biomarker Technologies Corporation, Beijing, China.

### Bioinformatics

The raw data were filtered primarily via Trimmomatic v0.33^35,80^. The primer sequences were identified and removed via Cutadapt v1.9.1^81^. The remaining high-quality sequences were processed via QIIME 2 (v. 2020.6)^74,82^, with the reads being processed and applied to the DADA2 pipeline for the assignment of amplicon sequence variants (ASVs)^83^. The sequences were classified into ASVs via the naive Bayesian classifier-based method, with a 0.005% conservative threshold for ASV filtration^84^. The UNITE 8.0 database^85^ was searched to determine the taxonomic classification of each fungal ASV. Fungal samples were rarefied to 45,210 sequences per sample before downstream analysis. The composition and diversity of the soil fungal community were analyzed via ASV tables. The ecological functions of the fungi were assigned via FUNGuild v1.0 with the confidence rankings of highly probable and probable levels^25^. This allowed us to explicitly correlate fungal functional potential with plant AGB.

### Statistics and reproducibility

Fungal Chao1 richness was calculated for each of the 288 soil samples. In order to avoid sampling biases, all information from 2 replicate plots on each small island or 4 replicate plots on each large island was averaged to obtain island-level estimates of soil physicochemical properties, fungal communities, and plant AGB (Supplementary Data 1). Average estimates of each target plant species on each island were used on statistical analysis, such as Spearman correlation, linear mixed effect model, and structural equation modeling. One-way ANOVA was conducted to determine the significance of mycorrhizal type and leaf habit type (i.e., evergreen broad-leaved, deciduous broad-leaved, coniferous) on the responses of AGB and fungal richness in rhizosphere soil, and Tukey’s HSD test was used for multiple comparisons across plant traits. The effects of variations in fungal community composition on differences by plant species and island area were visualized by non-metric multidimensional scaling (NMDS) using the vegan package v2.6-2^86^, which is based on Bray‒Curtis dissimilarity matrices. Before the following analysis, the island area, island isolation, and plant AGB data were log transformed to maintain normality.

To test the first hypothesis, Spearman correlation analysis was used to check the relationships between island spatial (island area and isolation) and soil fungal variables as well as plant AGB. The rhizosphere fungal variables and AGB within each plant species were standardized separately to remove plant species effects. To test the second hypothesis, a linear mixed effect model was performed via the lmerTest package v.3.1-3^87^ to conduct analyses of variance for plant AGB at the individual (ABIN), 400 m^2^ plot (ABP), and island (ABP) scales, with island spatial variables, soil environmental variables, and fungal alpha diversity as fixed factors and plant species as random factors. Principal component analysis (PCA) was performed on 8 soil physicochemical traits to reduce the number of environmental variables. All fixed factors were standardized to quantify their relative effects on plant AGB. The influence of plant species on the relationships between island variables and fungal alpha diversity was ruled out during the selection of the optimum linear mixed effect model. To test the third hypothesis, structural equation modeling was performed via the piecewiseSEM package v4.0.5^88^ to estimate the synthetic effect pathways of island spatial, soil P, and symbiotic fungal richness on plant AGB. Rhizosphere fungal variables and AGB within each plant species were standardized separately to remove plant species effects. All other variables were standardized to meet the assumptions of normality and homogeneity of variance. All the statistical analyses and visualizations were performed in R software v4.2.2^89^.

### Reporting summary

Further information on research design is available in the Nature Portfolio Reporting Summary linked to this article.

## Supplementary information

Supplementary Information

Description of Additional Supplementary Files

Supplementary Data

Reporting Summary

## Data Availability

The raw sequences of fungi were submitted to the NCBI-SRA and are available under the accession number PRJNA1120522. All the other data generated in this study are provided in the supplementary files.

## Code availability

All R packages and software versions needed to reproduce the results are presented in the Methods.
