# Supplementary material for: Low soil phosphorus and high symbiotic fungal richness inhibits plant aboveground biomass in fragmented forests in China
Source: Commun Biol. 2025 Nov 18;8:1598. doi: 10.1038/s42003-025-08978-w (PMC12627806; doi:10.1038/s42003-025-08978-w)
Supplement: Supplementary file 1 — Supplementary Information [file 42003_2025_8978_MOESM1_ESM.doc]

Supplementary Information for:

**Low soil phosphorus and high symbiotic fungal richness inhibits plant aboveground biomass in fragmented forests in China**

Jing Guo^1*^, Liying Chu^2^, Xuying Ye^3^, William L. King^4^, Jianbing Shao^5^, Zhonghan Wang^2^, Jinliang Liu^6^, Chuwen Chen^1^, Mingjian Yu^2,7*^

^1^College of Landscape Architecture, Zhejiang A&F University, Hangzhou, China

^2^College of Life Sciences, Zhejiang University, Hangzhou, China

^3^Forestry Bureau of Chun’an County, Hangzhou, China

^4^School of Biological Sciences, University of Southampton, Southampton, UK

^5^Xin’an River Ecological Development Group Corporation in Chun’an County, Hangzhou, China

^6^College of Life and Environmental Science, Wenzhou University, Wenzhou, China

^7^College of Forestry and Biotechnology, Zhejiang A&F University, Hangzhou, China

*Correspondence:

Jing Guo

Email: guojing@zafu.edu.cn

Mingjian Yu

Email: fishmj@zju.edu.cn


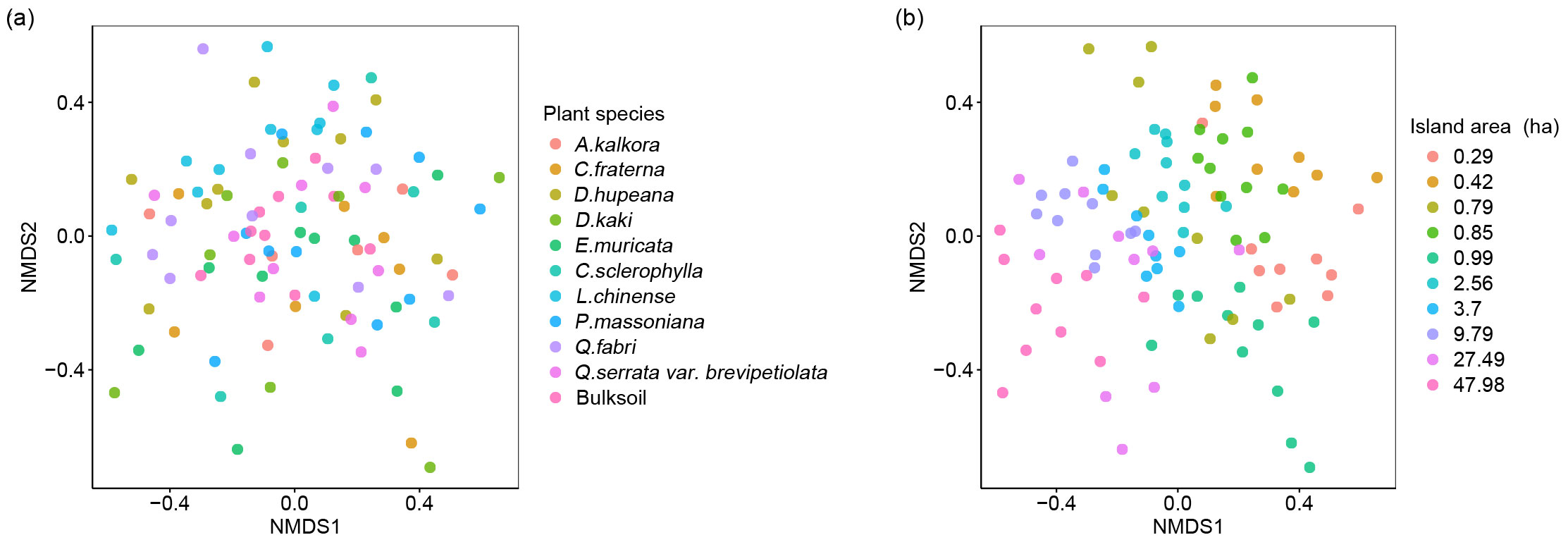


**Supplementary Fig. 1** Ordination of soil fungal samples via two-dimensional nonmetric multidimensional scaling (NMDS). In Panel (a), samples are coloured according to the rhizosphere of different plant species or the bulk soil. In Panel (b), the samples are coloured according to the island area.

**
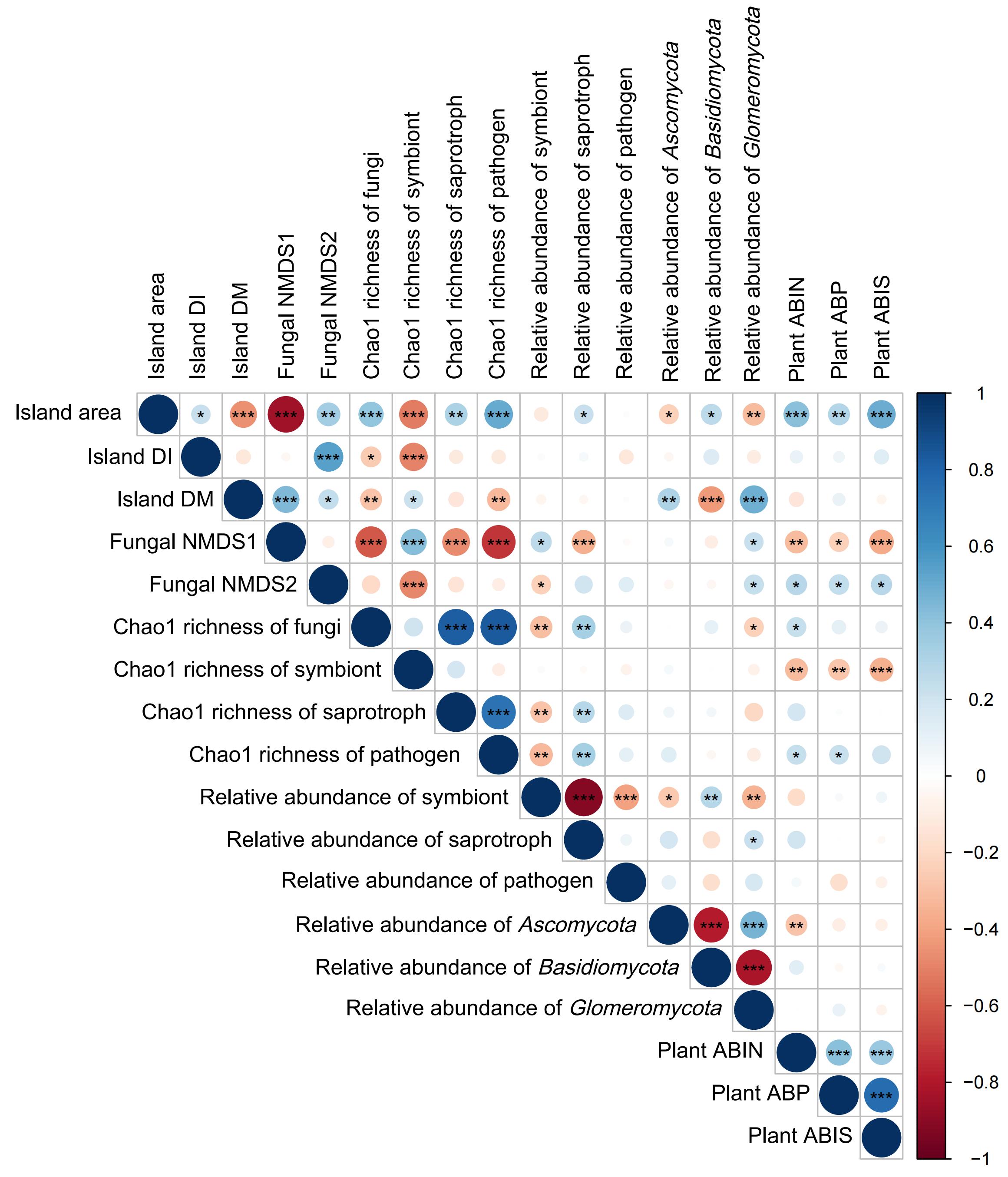
**

**Supplementary Fig. 2** Spearman correlations between island spatial and fungal community traits and plant AGB. *, *P* < 0.05; **, *P* < 0.01; ***, *P* < 0.001. DI, distance to the nearest island; and DM, distance to the mainland. Relative abundance of *Ascomycota*, *Basidiomycota* and *Glomeromycota* represent the relative abundance of these phyla within the symbiotic fungal group. ABIN, average aboveground biomass (AGB) per sampled individual plant; ABP, average AGB per individual of the target plant species in the 400 m^2^ plot; and ABIS, average AGB per individual of the target plant species on the entire island.


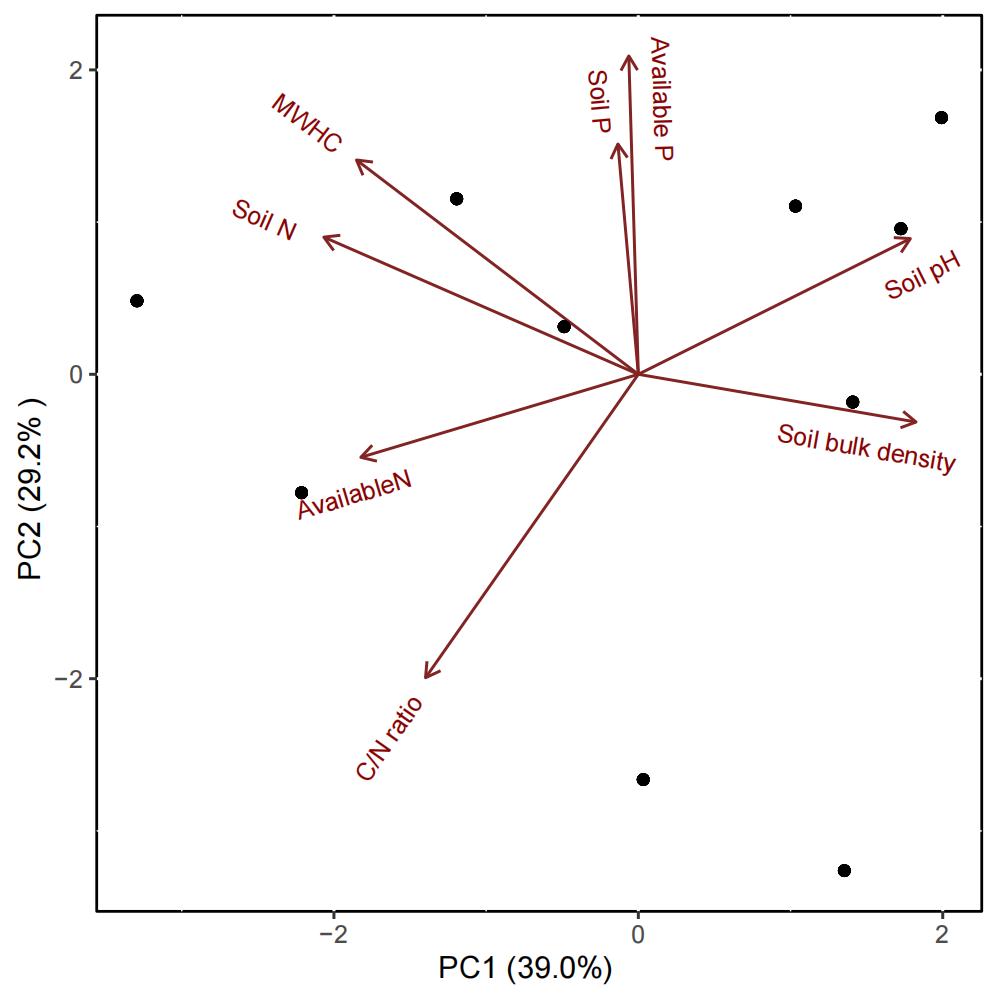


**Supplementary Fig. 3** Principal component analysis (PCA) of 8 soil physicochemical properties. MWHC, soil maximum water holding capacity.


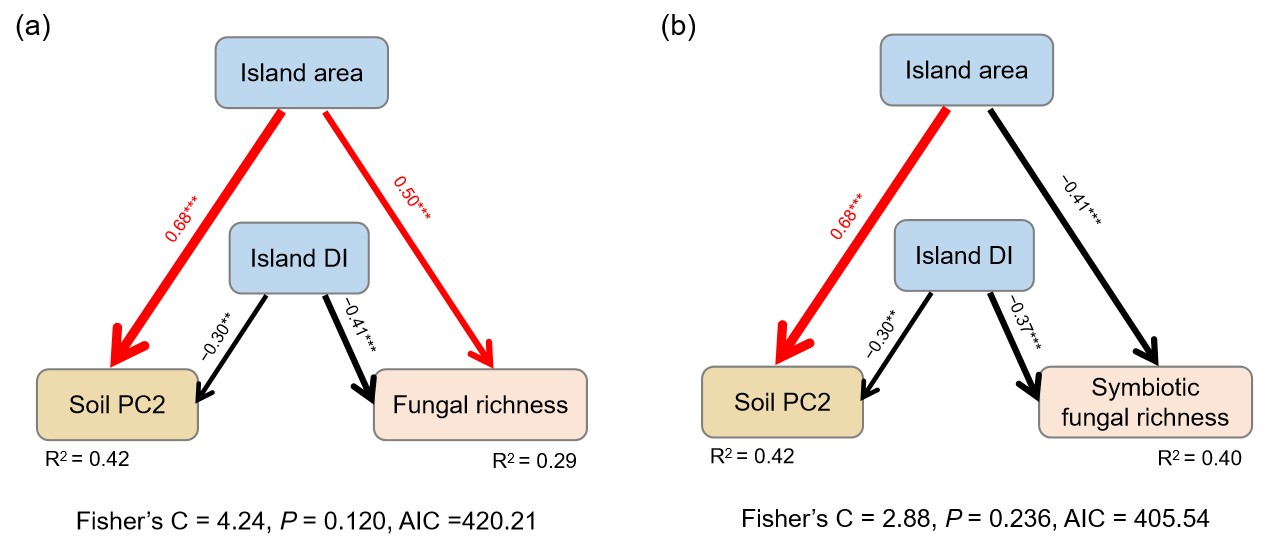


**Supplementary Fig. 4** Structural equation model showing the pathways through which island spatial traits influence fungal richness in the plant rhizosphere. Panel (a) shows the influence pathway of island spatial traits on overall fungal richness. Panel (b) shows the influence pathway of island spatial traits on symbiotic fungal richness. The percentages associated with the response variables represent the variance explained by the model. DI, distance to the nearest island. Red arrows indicate positive relationships and black arrows indicate negative relationships. The values associated with the arrows indicate standardized path coefficients. *, *P* < 0.05; **, *P* < 0.01; and ***, *P* < 0.001.

**Supplementary Table 1** Parameters of a power function equation relating tree height (*H*, m) and diameter at breast height (*D*, cm) for the targeted species in the TIL region in this study. The equation was *H* = *aD^b^* and was fitted through linear regression on log-transformed data. *CF* is a correction factor. The final model for the height estimate is H = *CF*×*a*D*^b^*.

| **Species** | ***a*** | ***b*** | **Adj.r^2^** | ***CF*** |
| --- | --- | --- | --- | --- |
| *Pinus massoniana* | 2.31514 | 0.56458 | 0.9118 | 1.010182 |
| *Quercus serrata* var. *brevipetiolata* | 2.566133 | 0.51193 | 0.8361 | 1.012184 |
| *Loropetalum chinense* | 3.038093 | 0.48234 | 0.8905 | 1.006925 |
| *Camellia fraterna* | 2.030943 | 0.66278 | 0.9036 | 1.009085 |
| *Eurya muricata* | 2.304284 | 0.59205 | 0.854 | 1.011274 |
| Other species | 2.353706 | 0.564976 | 0.9148 | 1.015602 |

**Supplementary Table 2** Allometric equations for aboveground biomass used in this study.

| **Species** | **Allometric equations** | **R^2^** |
| --- | --- | --- |
| *Pinus massoniana* | AGB = 0.13596×(D^2^×H) ^0.79^ | 0.91 |
| *Loropetalum chinense* | AGB = 0.1599×D^2.35119^ | 0.99 |
| *Quercus fabri* | AGB = 0.1199×(D^2^×H) ^0.8509^ | 0.99 |
| *Quercus serrata* var. *brevipetiolata* | AGB = 0.1199×(D^2^×H) ^0.8509^ | 0.99 |
| Other species | AGB = 0.09459×(D^2^×H) ^0.87^ | 0.91 |

AGB: aboveground dry biomass (kg), D: diameter at breast height (cm), H: plant height (m).

**Supplementary Table 3** Linear regression between fungal richness (dependent variable, y axis) and island size (independent variable, x axis, log (ha)).

| **Plant and soil** | **Mycorrhizal type** | **Leaf habit** | **Fungi** | | **Symbionts** | | **Saprotrophs** | | **Pathogens** | |
| --- | --- | --- | --- | --- | --- | --- | --- | --- | --- | --- |
|  |  |  | **Equation** | **R** | **Equation** | **R** | **Equation** | **R** | **Equation** | **R** |
| *Albizia kalkora* | AM | Deciduous broad-leaved | y = -7.29x+445 | 0.20 | y = -9.83x+ 43.9 | -0.94** | y = -9.80x+48.0 | 0.31 | y = -1.45x+11.7 | 0.37 |
| *Camellia fraterna* | AM | Deciduous broad-leaved | y = 91.0x+402 | 0.71† | y = -5.86x+44.3 | -0.50 | y = 5.97x+43.1 | 0.25 | y = 4.34x+7.29 | 0.66 |
| *Dalbergia hupeana* | AM | Deciduous broad-leaved | y = 86.5x+465 | 0.66* | y = -5.05x+39.8 | -0.29 | y = 3.84x+53.6 | 0.51 | y = 2.87x+11.9 | 0.50 |
| *Diospyros kaki* | AM | Deciduous broad-leaved | y = -13.5+504 | -0.14 | y = -7.24x+44.8 | -0.66† | y = -1.74x+56.7 | -0.23 | y = 0.78x+13.5 | -0.16 |
| *Eurya muricata* | AM | Evergreen broad-leaved | y = 25.3x+441 | 0.31 | y = -7.85x+44.7 | -0.50 | y = 3.04x+46.8 | 0.42 | y = 2.61x+8.58 | 0.42 |
| *Castanopsis sclerophylla* | ECM | Evergreen broad-leaved | y = 47.6x+405 | 0.46 | y = -9.80x+42.4 | -0.59 | y = 1.25x+45.6 | 0.07 | y = 5.03x+9.25 | 0.42 |
| *Loropetalum chinense* | ECM | Evergreen broad-leaved | y = 89.5x+424 | 0.75* | y = -4.43x+39.9 | -0.28 | y = 5.02x+49.8 | 0.50 | y = 5.87x+9.63 | 0.87*** |
| *Pinus massoniana* | ECM | Coniferous | y = 49.3x+372 | 0.55† | y = -4.15x+38.0 | -0.45 | y = 3.34x+42.8 | 0.44 | y = 2.48x+7.83 | 0.62† |
| *Quercus fabri* | ECM | Deciduous broad-leaved | y = 67.3x+467 | 0.41 | y = -8.94x+42.2 | -0.71* | y = 5.61x+49.2 | 0.39 | y = 4.76x+11.9 | 0.66* |
| *Quercus serrata* var. *brevipetiolata* | ECM | Deciduous broad-leaved | y = 13.1x+476 | 0.21 | y = -6.77x+39.6 | -0.43 | y = 2.70x+52.6 | 0.38 | y = 2.74x+11.6 | 0.58† |
| Bulk soil | / | / | y = 114x+581 | 0.72* | y = -3.21x+47.4 | -0.21 | y = 14.2x+68.2 | 0.63* | y = 1.47x+15.0 | 0.20 |

†, *P* < 0.1; *, *P* < 0.05; **, *P* < 0.01.

**Supplementary Table 4** The first two axes values of the principal component analysis (PC1 and PC2) for 8 soil environmental factors. The soil environmental factors included the soil pH, soil bulk density, MWHC, soil N, C/N ratio, available N, soil P and available P. MWHC, maximum water holding capacity of the soil.

| **Island ID** | **PC1** | **PC2** |
| --- | --- | --- |
| I1 | -0.37312 | 0.41645 |
| I2 | 0.62264 | 0.60901 |
| I3 | -0.69161 | -0.28103 |
| I4 | -0.15239 | 0.113 |
| I5 | 0.42309 | -1.17779 |
| I6 | 0.01011 | -0.96171 |
| I7 | 0.53923 | 0.34556 |
| I8 | -1.02974 | 0.17448 |
| I9 | 0.32284 | 0.39907 |
| I10 | 0.4403 | -0.06594 |
