# Supplementary material for: Low soil phosphorus and high symbiotic fungal richness inhibits plant aboveground biomass in fragmented forests in China
Source: Commun Biol. 2025 Nov 18;8:1598. doi: 10.1038/s42003-025-08978-w (PMC12627806; doi:10.1038/s42003-025-08978-w)
Supplement: Supplementary file 2 — Description of Additional Supplementary Files [file 42003_2025_8978_MOESM2_ESM.pdf]

Description of Additional Supplementary Files

File name- Supplementary Data 1

File description – Data that used to plot Fig.s 2-4 are provided in the Supplementary Data 1
