# Supplementary material for: Low soil phosphorus and high symbiotic fungal richness inhibits plant aboveground biomass in fragmented forests in China
Source: Commun Biol. 2025 Nov 18;8:1598. doi: 10.1038/s42003-025-08978-w (PMC12627806; doi:10.1038/s42003-025-08978-w)
Supplement: Supplementary file 4 — Reporting Summary [file 42003_2025_8978_MOESM4_ESM.pdf]

nature portfolio

Corresponding author(s): Dr. Jing Guo

Last updated by author(s): Sep 13, 2025

Reporting Summary

Nature Portfolio wishes to improve the reproducibility of the work that we publish. This form provides structure for consistency and transparency in reporting. For further information on Nature Portfolio policies, see our Editorial Policies and the Editorial Policy Checklist.

Statistics

For all statistical analyses, confirm that the following items are present in the figure legend, table legend, main text, or Methods section.

n/a	Confirmed	
☐	☒	The exact sample size (*n*) for each experimental group/condition, given as a discrete number and unit of measurement
☐	☒	A statement on whether measurements were taken from distinct samples or whether the same sample was measured repeatedly
☐	☒	The statistical test(s) used AND whether they are one- or two-sided<br>*Only common tests should be described solely by name; describe more complex techniques in the Methods section.*
☐	☒	A description of all covariates tested
☐	☒	A description of any assumptions or corrections, such as tests of normality and adjustment for multiple comparisons
☐	☒	A full description of the statistical parameters including central tendency (e.g. means) or other basic estimates (e.g. regression coefficient) AND variation (e.g. standard deviation) or associated estimates of uncertainty (e.g. confidence intervals)
☐	☒	For null hypothesis testing, the test statistic (e.g. *F*, *t*, *r*) with confidence intervals, effect sizes, degrees of freedom and *P* value noted<br>*Give P values as exact values whenever suitable.*
☒	☐	For Bayesian analysis, information on the choice of priors and Markov chain Monte Carlo settings
☐	☒	For hierarchical and complex designs, identification of the appropriate level for tests and full reporting of outcomes
☐	☒	Estimates of effect sizes (e.g. Cohen's *d*, Pearson's *r*), indicating how they were calculated

Our web collection on statistics for biologists contains articles on many of the points above.

Software and code

Policy information about availability of computer code

Data collection	Roots and soil were sampled from April 20th-May 14th. Rhizosphere soil around each plant were pooled together and mixed to form one sample. the purified, pooled PCR products were subsequently sequenced on the Illumina NovaSeq 6000PE250 platform.
Data analysis	All the statistical analyes and visualizations were performed in R software v4.2.2.

For manuscripts utilizing custom algorithms or software that are central to the research but not yet described in published literature, software must be made available to editors and reviewers. We strongly encourage code deposition in a community repository (e.g. GitHub). See the Nature Portfolio guidelines for submitting code & software for further information.

Data

Policy information about availability of data

All manuscripts must include a data availability statement. This statement should provide the following information, where applicable:

- Accession codes, unique identifiers, or web links for publicly available datasets
- A description of any restrictions on data availability
- For clinical datasets or third party data, please ensure that the statement adheres to our policy

The raw sequences of fungi were submitted to the NCBI-SRA and are available under the accession number PRJNA1120522. All the other data generated in this study are provided in the supplementary files.

Research involving human participants, their data, or biological material

Policy information about studies with human participants or human data. See also policy information about sex, gender (identity/presentation), and sexual orientation and race, ethnicity and racism.

Reporting on sex and gender	n/a
Reporting on race, ethnicity, or other socially relevant groupings	n/a
Population characteristics	n/a
Recruitment	n/a
Ethics oversight	n/a

Note that full information on the approval of the study protocol must also be provided in the manuscript.

Field-specific reporting

Please select the one below that is the best fit for your research. If you are not sure, read the appropriate sections before making your selection.

☐ Life sciences ☐ Behavioural & social sciences ☒ Ecological, evolutionary & environmental sciences

For a reference copy of the document with all sections, see nature.com/documents/nr-reporting-summary-flat.pdf

Ecological, evolutionary & environmental sciences study design

All studies must disclose on these points even when the disclosure is negative.

Study description	Ten islands ranging in area and ten woody plant species were chosen for this study. In total, 30 plots were established on all islands.
Research sample	Ten woody plant species were chosen for this study, with five associated with arbuscular mycorrhizal (AM) fungi and five associated with ectomycorrhizal (ECM) fungi.
Sampling strategy	One representative plant of each target species in a plot was selected for rhizosphere fungal estimation. Rhizosphere soil around each plant were pooled together and mixed to form one sample. A total of 288 soil samples were collected.
Data collection	All the statistical analyses and visualizations were performed in R software v4.2.2.
Timing and spatial scale	Roots and soil were sampled from April 20th-May 14th, 2022.
Data exclusions	n/a
Reproducibility	Ten woody plant species were chosen for this study.
Randomization	In order to avoild sampling biases, all informatio from 2 replicate plots on each small island or 4 replicate plots on each large island was averaged to obtain island-level estimates of soil physicochemical properties, fungal communities, and plant AGB.
Blinding	n/a

Did the study involve field work? ☒ Yes ☐ No

Field work, collection and transport

Field conditions	Roots and soil were sampled on fine weather from April 20th-May 14th, 2022.
Location	The study was conducted at the Thousand Island Lake (TIL) in Zhejiang Province, eastern China (Fig.1).
Access & import/export	Soil samples were collected and stored at -80? for DNA extraction and ???sequencing.?
Disturbance	n/a

Reporting for specific materials, systems and methods

We require information from authors about some types of materials, experimental systems and methods used in many studies. Here, indicate whether each material, system or method listed is relevant to your study. If you are not sure if a list item applies to your research, read the appropriate section before selecting a response.

Materials & experimental systems

n/a	Involved in the study	
☒	☐	Antibodies
☒	☐	Eukaryotic cell lines
☒	☐	Palaeontology and archaeology
☐	☒	Animals and other organisms
☒	☐	Clinical data
☒	☐	Dual use research of concern
☒	☐	Plants

Methods

n/a	Involved in the study	
☐	☒	ChIP-seq
☒	☐	Flow cytometry
☒	☐	MRI-based neuroimaging

Animals and other research organisms

Policy information about studies involving animals; ARRIVE guidelines recommended for reporting animal research, and Sex and Gender in Research

Laboratory animals	n/a
Wild animals	n/a
Reporting on sex	n/a
Field-collected samples	Three to four root clusters were collected from shallow soil around the trunk of each selected plant within a 2 m radius. After gently shaking,the soil still adhering to the fine-root branches was considered as rhizosphere soil. Moreover, five 0-10 cm deep soil cores were collected, mixed, and passed through a seive of 2 mm mesh size to form on e bulk soil sample. A total of 288 soil samples were collected and stored at -80 for DNA extraction and sequenccing.
Ethics oversight	No ethical approvalor guidance was required because we measure soil fungi.

Note that full information on the approval of the study protocol must also be provided in the manuscript.

Plants

Seed stocks	n/a
Novel plant genotypes	n/a
Authentication	n/a

ChIP-seq

Data deposition

☒ Confirm that both raw and final processed data have been deposited in a public database such as GEO.

☒ Confirm that you have deposited or provided access to graph files (e.g. BED files) for the called peaks.

Data access links<br>*May remain private before publication.*	The raw sequences of fungi were submitted to the NCBI-SRA and are available under the accession number PRJNA1120522. All the other data generated in this study are provided in the supplementary files.
Files in database submission	Supplemental files, Supplementary Data1
Genome browser session<br>(e.g. UCSC)	

Provide a link to an anonymized genome browser session for "Initial submission" and "Revised version" documents only, to enable peer review. Write "no longer applicable" for "Final submission" documents.

Methodology

Replicates	Ten islands ranging in area from 0.29-47.98 ha, and ten woody plant pecies were chosen for this study. Ultimately, two replicate plots were established on each small island. On each large island, four replicateplots were estabilished, with two plots on the edge and another two plots in the interior of the island.
Sequencing depth	The sequences were classified into ASVs via the naivve Bayesian classifier-based method, with a 0.005% conservative threshold for ASV filtration.
Antibodies	n/a
Peak calling parameters	n/a
Data quality	Fungal samples were rarefied to 45210 sequences per sample before downstream analysis.
Software	The raw data were filtered primarily via Trimmomatic v0.33. The primer sequences were identified and removed via Cutadapt v1.9.1. The remaining high-quality sequences were processed via QIIME 2(v2020.6). All the statistical analyses and visualizations were performed in R software v4,2,2.
